# Local Treatment of Acne

**Published:** 1878-06

**Authors:** 


Local Treatment of Acne.—Dr. Robert Liveing, in the London *Lancet,* advises the following plan as generally successful: (1.) The face should be steamed every night by holding it over a basin of hot water for a few minutes. (2.) The skin should then be well rubbed for five or ten minutes with soap and flannel, or a soft nail brush may be used with advantage where the skin will bear it; the soap should then be sponged off with warm water. (3.) When the face has been dried, a lotion composed of half an ounce of precipitated sulphur, two drachms of glycerine, one ounce of spirits of wine, three ounces each of lime water and rose water, should freely be applied and allowed to remain all night. Add ether to the lotion, if the skin is greasy. Sometimes an ointment—hypochloride of sulphur ℈ iss., carbonate of potash gr. x., oil of bitter almonds gtt. x., and lard ℥ i., or sulphur ointment ℥ iij., with vaseline ℥ v.—is better than the lotion. In either case leave the application on all night, and in the morning let it be washed off with warm oatmeal and water or weak gruel.

